# The Impact of Implementation Fidelity of a School-Based Multi-Component Smoking Prevention Intervention on Vocational Students’ Smoking Behavior: A Cluster-Randomized Controlled Trial

**DOI:** 10.1007/s11121-024-01712-8

**Published:** 2024-08-02

**Authors:** Marie Pil Jensen, Rikke Fredenslund Krølner, Lau Caspar Thygesen, Lisbeth Lund, Susan Andersen

**Affiliations:** The National Institute of Public Health, University of Southern Denmark, Studiestræde 6, 1455 Copenhagen K, Denmark

**Keywords:** Process evaluation, Complex interventions, School tobacco policies, Older adolescents, School-based smoking prevention, Smoking cessation

## Abstract

Preventing young people’s cigarette smoking is a major public health priority, and smoking is especially prevalent in vocational schools. Well-enforced comprehensive school tobacco policies accompanied by preventive efforts show potential to reduce smoking, but the implementation process is crucial to achieve the intended effect. We investigate whether and how implementation fidelity of a multi-component smoking prevention intervention impacted student smoking outcomes after 4–5 months among students in Danish vocational education and training (national age range 15–65 years, mean 25.6) and preparatory basic education (national age range 15–25 years, mean 17.6) institutions using questionnaire data from a cluster-RCT. The intervention included a smoke-free school hours policy, educational curriculum, and class competition. We calculated an overall implementation fidelity measure combining staff-reported school-level delivery (fidelity) and student-reported receipt (participation, responsiveness), and used multilevel regression models to analyze associations with smoking outcomes (smoking daily, regularly, and during school hours). We supplemented the analysis with restricted cubic spline regression. Additionally, we stratified the analyses by school types and analyzed associations between implementation fidelity of the separate intervention components and smoking outcomes. High implementation was associated with lower odds of regular smoking (OR: 0.37, 95% CI: 0.18–0.78) and smoking during school hours, but not daily smoking, and these associations varied between the school settings. When analyzed separately, implementation fidelity of the components did not affect the outcomes significantly. Our findings underline the need to support the implementation process of school tobacco policy interventions to ensure the intended effects of reducing students’ smoking.

**Supplementary Information:**

The online version contains supplementary material available at 10.1007/s11121-024-01712-8.

## Background

Cigarette smoking can cause a range of adverse health effects such as cancer and premature death (Surgeon General, 2014). The global prevalence of smoking among 15–24-year-olds is 20% among males and 5% among females, and most people who smoke initiate between 14 and 25 years of age (Reitsma et al., 2021). Early onset of smoking increases the risk of nicotine dependence later in life (Breslau et al., 1993; Kendler et al., 2013), and smoking across the life course (being a life-long smoker with initiation by the age of 18) has been shown to increase the risk threefold of all-cause mortality by the age of 60 (Clennell et al., 2008). Thus, young people’s cigarette smoking is a major public health priority worldwide. In Denmark, 9% of 15–29-year-olds smoke cigarettes daily (Petersen et al., 2022). This is also the prevalence of daily smoking among students in academic high schools (Pisinger et al., 2019), but the prevalence is 29% among students in vocational education and training (vocational school) (Ringgaard et al., 2020), a type of upper secondary education for skilled professions. This educational disparity in daily smoking is a consistent trend throughout Europe (de Looze et al., 2013). Studies have indicated a strong association between low educational achievement, a disadvantaged socioeconomic position, and elevated rates of smoking (Hiscock et al., 2012; Hanson and Chen, 2007; de Looze et al., 2013). Consequently, addressing smoking prevention among young individuals in low socioeconomic positions is crucial.

School-based smoking prevention interventions have been delivered to students for more than 40 years with varying degrees of effectiveness (Thomas et al., 2013), and interventions aimed to develop social skills and competence in combination with training to resist the social pressure of smoking appear to be most effective (Thomas et al., 2013). Universal school-based interventions are effective in reducing smoking behavior among young people up to 18 years (MacArthur et al., 2018). However, interventions focused on young people of early adulthood (18–25 years) are reported less frequently although smoking initiation and transition to daily smoking have been increasing in this age group in recent decades (Barrington-Trimis et al., 2020). In upper secondary school settings (e.g., high schools or vocational schools equivalent to International Standard Classification of Education level 3), school tobacco policies (STPs) have commonly been employed as tobacco control (Galanti et al., 2014; Mélard et al., 2020). Still, the evidence of their effectiveness is inconclusive due to a lack of experimental and longitudinal studies and definitions of policy dimensions (Galanti et al., 2014). More recently, however, longitudinal and quasi-experimental studies suggest a link between STPs and decreased smoking (Azagba et al., 2015; Källmén et al., 2020; Mélard et al., 2020). Another study found no long-term effect of an outdoor smoking ban (Rozema et al., 2018). Evidence suggests that STPs are associated with less smoking among upper secondary students when they include clear rules and strict and consistent enforcement, are combined with preventive and educational components, and are comprehensive with regard to, e.g., the policy’s targeted subjects, locations, types of tobacco products, and school activities (Galanti et al., 2014; Schreuders et al., 2017). Less comprehensive STPs, such as the prohibition of smoking on school premises only, have been shown to displace smoking (Galanti et al., 2014; Hewer et al., 2022; Mélard et al., 2020). Such policies promote the visibility of smoking outside school’s ground and thereby increase the perceived acceptability of smoking in the school environment (Coppo et al., 2014; Galanti et al., 2014; Mélard et al., 2020). Thus, a comprehensive policy such as smoke-free school hours, i.e., the prohibition of smoking for all students, staff, and visitors on or outside school premises during school hours, might have more potential to prevent smoking in upper secondary school settings. As for vocational schools, few experimental studies on potential strategies to prevent or reduce smoking have been conducted (Hjort et al., 2022; Sussman et al., 2014). Therefore, we developed an intervention named “Focus” aiming to reduce smoking at Danish vocational schools. The intervention entailed the introduction and enforcement of a comprehensive tobacco policy (smoke-free school hours) and multiple components targeted at individual, interpersonal, and environmental determinants of students’ smoking (Andersen et al., 2023).

The effectiveness of interventions depends on their implementation process (Durlak & DuPre, 2008). Recent studies indicate that implementing STPs in secondary school settings across Europe and in Denmark is challenging (Hjort et al., 2021; Hoffmann et al., 2020; Schreuders et al., 2019). Barriers include a low prioritization of smoking prevention and policies, insufficient human resources to enforce policies, staff’s perception of resistance, and non-compliance to STPs predominately among persistently smoking and socially disadvantaged students, especially in vocational schools (Hoffmann et al., 2020). Additionally, implementation and enforcement of STPs may be hindered by staff perceptions; for example, some work has shown that staff express concern that enforcement interferes with positive student-staff relations (Hjort et al., 2021; Schreuders et al., 2019). Furthermore, aspects of the implementation process such as inconsistent enforcement might counteract the positive impacts of STPs on students’ smoking and cause reverse effects (Schreuders et al., 2017). These challenges underline the need to carefully assess the implementation process when estimating the effectiveness of STPs on students’ smoking. A crucial part of the implementation process is implementation fidelity, that is, the extent to which interventions are delivered as intended (Durlak & DuPre, 2008; Moore et al., 2015). Recent studies assessed the implementation fidelity of STPs in secondary schools (Rozema et al., 2018) and vocational schools (Hjort et al., 2022). However, the study in secondary schools lacked explicit conceptual underpinning of implementation fidelity (Rozema et al., 2018), and the study in vocational schools focused on implementation strategies to enhance implementation fidelity (Hjort et al., 2022). To our knowledge, no experimental studies have specifically investigated whether the extent of implementation of STPs in school settings of older adolescents and young adults is linked to reduced smoking.

The “Focus” intervention was evaluated in a cluster-randomized controlled trial in 2018–2020. We found no overall effectiveness of the intervention on students’ smoking (Kjeld et al., 2023). However, an intervention effect emerged in pre-planned subgroup and per protocol analyses, e.g., among students attending schools who delivered several components rather than parts of the intervention only (Kjeld et al., 2023) and students in a particular school type (social and healthcare vocational school). Moreover, the school types included in the RCT, which are characterized by different educational, organizational, and compositional features, implemented the intervention to varying extents (Jensen et al., 2023). This study further investigates the role of implementation on the intervention’s effectiveness for altering students’ smoking behavior using comprehensive measurements of implementation (Jensen et al., 2023) considering multiple aspects of the implementation process. Furthermore, identifying the “core” components of an intervention (Carroll et al., 2007; Durlak & DuPre, 2008; Linnan & Steckler, 2002; Skivington et al., 2021), i.e., elements that are essential for a program’s success (Blase & Fixsen, 2013; Carroll et al., 2007), is considered an important part of any evaluation of a new intervention (Carroll et al., 2007). It includes determining which components or features of an intervention that make a difference to its intended outcomes. Identifying essential components can, for example, enable the ability to strengthen and target implementation strategies to the most effective components (Blase & Fixsen, 2013) and support the scale-up or adaptation of an intervention to other settings to improve fit and acceptance without undermining the components known to drive its effectiveness (Blase & Fixsen, 2013).

This study aims to investigate whether the implementation fidelity of the multi-component “Focus” intervention (i.e., the extent to which it was delivered and received as intended) affected students’ smoking outcomes (daily smoking, regular smoking, and smoking during school hours) 4–5 months after baseline. The objectives were to investigate (1) the association between implementation fidelity of the total intervention and smoking outcomes, (2) differential effects between school types of total intervention implementation fidelity on smoking outcomes, and (3) the association between implementation fidelity of the separate intervention components and smoking outcomes.

## Methods

### Setting

The Danish Vocational Education and Training system (vocational schools) educates students for skilled professions through programs of varying duration, consisting of a school-based basic part and a main part that shifts between apprenticeships and school periods. The basic program is divided into two parts; the first part is mandatory for the youngest students who completed lower secondary school within the last two years upon enrolment, and all other students can begin at the second part. For this reason, students’ age spans from 15 to 65 (Ringgaard et al., 2020). Preparatory Basic Education (preparatory schools) is intended for young people aged 15 to 25 years old who are not in education or employment and need professional, social, or personal support and qualifications to gain employment opportunities or proceed to upper secondary education such as vocational school.

### Study Design and Data Collection

The intervention was evaluated in a cluster-randomized controlled trial and conducted over two periods of a school semester, August to December, in 2018 and 2019. Randomization was stratified by school types: social and healthcare vocational schools, technical and commercial vocational schools, and preparatory schools. Eight schools were allocated to the intervention arm and six to the control group (continuing with usual practice). Questionnaire data were collected among students, teachers, and principals at all schools at baseline and follow-up after 4–5 months. Student questionnaires addressed, e.g., background characteristics such as sociodemographic factors, and smoking-related questions (more details under outcome variables and statistical analyses). Additionally for intervention schools, student, teacher, and principal follow-up questionnaires contained questions about the implementation of the intervention components as part of the process evaluation (presented in detail under exposure variables). One preparatory school in the intervention group partially withdrew from the study after randomization due to organizational changes and heavy workload (Jensen et al., 2023). This school received reduced versions of the implementation questionnaires and was therefore not included in the present study. Details on the study design, recruitment, and randomization procedures are published elsewhere (Jakobsen et al., 2021; Kjeld et al., 2023).

### Intervention Components

The “Focus” intervention was developed in 2017–2018 based on the stages and steps of the Behavior Change Wheel (Michie et al., 2011) including the Capability, Opportunity, Motivation, and Behavior model and Theoretical Domains Framework (Andersen et al., 2023). The intervention entailed six components: (1) the introduction and enforcement of smoke free school hours, a comprehensive STP stating that students, staff, and visitors are not allowed to smoke during the school day on or off school premises; (2) a course organized by the Danish Cancer Society for two to four staff members in short motivational counselling about smoking; (3) an “edutainment" event at the beginning of the school year delivered by a professional actor with a combination of information and entertainment to inform about the harms of smoking and challenge common misperceptions; (4) a class-based educational curriculum of eight sessions to challenge attitudes and beliefs, stimulate reflection, and support social activities not related to smoking; (5) a class-based “quit-and-win” competition to increase students’ mutual support and motivation to abstain from or quit smoking including a prize for the winning class; and (6) access to telephone-based support and counselling for smoking cessation or how to handle a smoke-free school day for all students and staff provided by The National Quitline. Detailed descriptions of each intervention component are published elsewhere along with a description of the intervention development (Andersen et al., 2023).

### Exposure Variables

#### Conceptual Framework and Quantification Model

We operationalized implementation fidelity measurements based on Carroll et al.’s (2007) conceptual framework for implementation fidelity and a model to quantify implementation (Carroll et al., 2007; Ferm et al., 2018). The model distinguishes between implementation at the organizational level (delivery), i.e., the extent to which the organization delivered the intervention as intended, and implementation at the individual level (receipt), i.e., the extent to which each participant was exposed and responded to the intervention as intended. To assess delivery and receipt, explicit success criteria must be determined from the program theory and assumptions of the intervention, i.e., what is required for the intervention to be successfully delivered and received and therefore work as intended? Thus, pre-specifying measurable success criteria is an essential first step of the quantification model. Key concepts of the organizational level include content defined as the “active ingredients” delivered to the participants such as treatment, knowledge, or skills, and quality of delivery referring to the manner with which the intervention’s content is delivered. Key concepts of the individual level include participation in relation to the intervention components and participant responsiveness such as the motivation, satisfaction, acceptability, attitudes, or engagement of the participants in response to the intervention, which may determine the individuals’ uptake of the intervention and moderate the level of implementation fidelity achieved. The framework and model take account of how the concepts interact with each other (Carroll et al., 2007; Ferm et al., 2018). Thus, based on the concepts of content, quality, participation, and responsiveness, the model prescribes calculations of organizational-level fidelity and individual-level exposure and implementation (Ferm et al., 2018) which are presented in the following.

#### Operationalization of Implementation Fidelity Measurements

Following the model to quantify implementation (Ferm et al., 2018), we either selected existing or designed new questionnaire items to align with the criteria of the content, quality, participation, and responsiveness of each intervention component with response categories representing percentages of 0 (not implemented), 50 (acceptably implemented), or 100 (optimally implemented) (Supplementary Table 1) (Ferm et al., 2018; Jensen et al., 2023). These criteria were based on the program theory and discussions in the project group (Ferm et al., 2018; Jensen et al., 2023). After follow-up data collection, we assigned percentages for each school (content, quality) and student (participation, responsiveness). Finally, we used the assigned percentages to calculate each school’s fidelity to the components and each student’s exposure to and individual-level implementation of the components (Ferm et al., 2018). Supplementary Table S1 shows how each concept was measured for each component if applicable and available. For example, the delivery of the tobacco policy covered whether the management had introduced the policy at the beginning of the school year (content), communication of the policy at school (quality), availability of a written policy (quality), and policy enforcement (quality). The receipt covered students’ thoughts about the fairness that the school makes rules for smoking during the school day (responsiveness). Participation was not applicable for this component.

Individual-level implementation was calculated as the student’s exposure to the component multiplied by the student’s responsiveness to the component:

$${Individual\; level\; implementation\; (ILI)}^{Component\; X}{=Exposure}^{Student} \times {Responsiveness}^{Student}$$

where the exposure was calculated as the school-specific fidelity to the component (mean of content and quality of delivery) multiplied by the student’s participation in the component (where applicable). We calculated implementation of the total intervention as the mean percentage of individual-level implementation of each intervention component:

$${Individual\; level\; implementation\; (ILI)}^{Total\; intervention}=\frac{{ILI}^{Component\; 1}\;+\;\left[\dots \right]\;+\;{ILI}^{Component\; 5}}{5}$$

(Ferm et al., 2018).

We calculated tertiles of the individual-level implementation variables to create a categorical variable including a category for the control group (control, low implementation, medium implementation, and high implementation).

### Outcome Variables

Two binary outcome variables were calculated from smoking status (“Do you smoke cigarettes?”) measured in questionnaires at baseline and follow-up: daily smoking (“Yes, everyday” vs. “Yes, at least once a week”, “Yes, more seldom than every week”, or “No, I do not smoke at present”) and regular smoking (“Yes, everyday” or “Yes, at least once a week” vs. “Yes, more seldom than every week” or “No, I do not smoke at present”). We included a third outcome variable, smoking during school hours, calculated from follow-up questionnaires (“How often do you smoke during the school day (with others or alone)?”): “Every day” or “Several days a week” vs. “Rarer” or “Never”.

### Statistical Analyses

We included students from the intervention (*n* = 656) and control group (*n* = 596) who responded to the follow-up questionnaire. We excluded 73 students with insufficient implementation data, 66 students with missing data on sex or age, and 1 student with missing data on all smoking outcomes, resulting in a final study population of *n* = 1112 students. Descriptive statistics and multilevel logistic regression models were performed in SAS 9.4, and we present the results as odds ratios (OR) with 95% confidence interval (CI) and *p* values. *P* values less than 0.05 are considered statistically significant. To address objective 1, we used 2-level logistic regression models with the GLIMMIX procedure (students nested within schools). We adjusted the models for self-reported sex (male or female), age calculated from the date of birth, socioeconomic status (SES) measured as student-reported family occupational social class, school type (social and healthcare, technical or commercial, and preparatory), and the outcome variable at baseline. The models with smoking during school hours as the outcome were adjusted for baseline regular smoking. We included school as random effects to account for clustering. As the cut-points for low, medium, and high implementation based on tertiles were arbitrary, we supplemented the models with graphic representations of fully adjusted restricted cubic spline regression models performed in RStudio (R Core Team, 2023) to be able to analyze all data points of the continuous variable and their relations to the outcome variables (Gauthier et al., 2020). To address objective 2, we stratified the logistic regression models by school type (social and healthcare students vs. technical, commercial, and preparatory school students). To address objective 3, we used the tertiles-based categorical variables of student-level implementation of the school tobacco policy, educational curriculum, and class competition components as exposures in multilevel logistic regression models. We only tested these three components separately because we did not expect the edutainment session to have an independent effect on the smoking outcomes as it was a one-time event at the beginning of the school year to support and raise awareness of the tobacco policy. Further, we did not have individual-level implementation measurements of the staff course, and only very few students reported use of the smoking cessation support offer. We performed additional analyses to decompose the student-level implementation construct into school-level fidelity (of the total intervention) and student responsiveness (of the smoke-free school hours component) and analyzed these variables’ relationships with the smoking outcomes. Finally, we compared the sociodemographic characteristics of the study population with the excluded students to assess potential selection bias.

## Results

### Characteristics of the Study Population

Most students in the present study were from technical or commercial (48%) or social and healthcare (37%) vocational schools, while 15% were preparatory students (Table 1). The median age was 17 years, and most students had a middle-level SES of social class III to IV. Twenty-one percent of the students smoked daily at baseline, and 9% smoked weekly or less than weekly. Slightly more students in the intervention group smoked daily (25%) than in the control group (18%).

**Table 1:** Baseline characteristics of the study population

	Total (*n* = 1112)	Intervention group (*n* = 545)	Control group (*n* = 567)
	*n* (%)	*n* (%)	*n* (%)
**School type**			
Social and healthcare	415 (37.2)	208 (38.2)	207 (36.5)
Technical or commercial	534 (48.0)	272 (49.9)	262 (46.2)
Preparatory basic	163 (14.7)	65 (11.9)	98 (17.3)
**Age, years (median)**	17	17	17
**Sex**			
Females	558 (50.2)	276 (50.6)	282 (49.7)
Males	554 (49.8)	269 (49.4)	285 (50.3)
**Family occupational social class**			
High (I + II)	165 (14.8)	85 (15.6)	80 (14.1)
Middle (III + IV)	449 (40.4)	224 (41.1)	225 (39.7)
Low (V + VI)	265 (23.8)	130 (23.9)	135 (23.8)
Unclassifiable or missing	233 (21.0)	106 (19.5)	127 (22.4)
**Smoking status**			
Daily smoking	237 (21.3)	136 (25.0)	101 (17.8)
Weekly smoking	33 (3.0)	15 (2.8)	18 (3.2)
Less than weekly smoking	71 (6.4)	39 (7.2)	32 (5.6)
Not smoking at present	583 (52.4)	292 (53.6)	291 (51.3)
Missing	188 (16.9)	63 (11.6)	125 (22.1)

### Effects of Implementation Fidelity of the Total Intervention on Smoking Outcomes

**Fig. 1:**
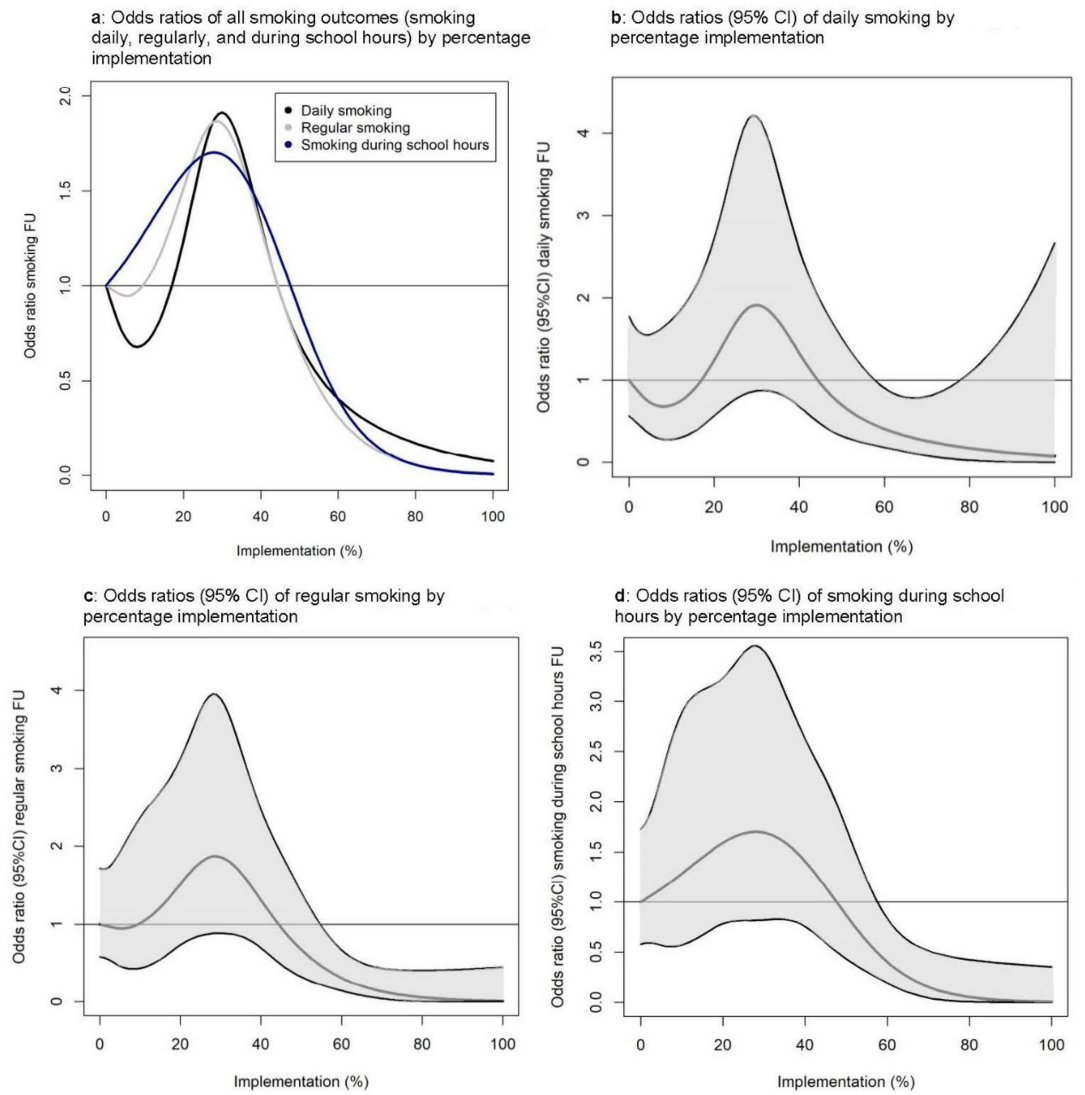
Odds ratios of smoking outcomes by level of implementation fidelity modelled from restricted cubic spline regression analysis

The student-level implementation scores were divided into tertiles: 32% of students had a low implementation level (0–23.3%), 33% of students had a medium implementation level (23.5–46.0%), and 35% of students had a high implementation level (46.5–100%) (Table 2). High implementation of the total intervention was associated with lower odds of regular smoking (OR 0.37, 95% CI 0.18–0.78), but not associated with daily smoking and smoking during school hours though the estimates were in the same direction (Table 2). The spline regression analyses (Fig. 1), however, showed that implementation levels between 60 and 80% were significantly associated with lower odds of daily smoking though insignificant at levels between 80 and 100% (Fig. 1b). Moreover, students with implementation levels of 60% or higher had significantly lower odds of regular smoking and smoking during school hours (Fig. 1c, d). Students with medium implementation had increased odds of all outcomes (Table 2) though not significantly, peaking at around 30% implementation (Fig. 1).

**Table 2:** Odds ratios (OR) of smoking daily, regularly, and during school hours by low, medium, and high implementation (low 0–23.3%, medium 23.5–46.0%, and high 46.5–100% (tertiles of continuous variable)) of the total intervention compared to students in the control group. All students and stratified by school type

		Daily smoking		Regular smoking		Smoking during school hours^a^	
	*n*	OR (95% CI) Unadjusted	OR (95% CI) Adjusted^b^	OR (95% CI) Unadjusted	OR (95% CI) Adjusted^b^	OR (95% CI) Unadjusted	OR (95% CI) Adjusted^b^
**All students**	1112						
Control (ref)	567	1	1	1	1	1	1
Low implementation	175	1.40 (0.67–2.95)	0.84 (0.34–2.07)	1.39 (0.74–2.62)	1.26 (0.68–2.34)	1.55 (0.86–2.80)	1.32 (0.60–2.92)
Medium implementation	181	1.76 (0.87–3.56)	1.63 (0.69–3.87)	1.75 (0.96–3.19)	1.72 (0.95–3.12)	1.83 (1.05–3.19)	1.66 (0.77–3.56)
High implementation	189	0.61 (0.29–1.28)	0.56 (0.21–1.49)	0.54 (0.28–1.03)	0.37 (0.18–0.78)	0.54 (0.29–1.00)	0.66 (0.20–1.13)
* p Value*		0.0005	0.070	< 0.0001	0.002	< 0.0001	0.009
**Social and healthcare students**	415						
Control (ref)	207	1	1	1	1	1	1
Low/medium^c^ impl	85	1.78 (0.92–3.46)	0.59 (0.13–2.79)	1.73 (0.76–3.93)	0.92 (0.20–4.14)	1.48 (0.88–2.48)	0.68 (0.30–1.54)
High implementation	123	0.57 (0.29–1.11)	0.38 (0.08–1.81)	0.49 (0.22–1.13)	0.28 (0.06–1.34)	0.39 (0.23–0.69)	0.25 (0.10–0.58)
* p Value*		0.001	0.435	0.0004	0.103	0.0002	0.006
**Technical, commercial, and preparatory students**	697						
Control (ref)	360	1	1	1	1	1	1
Low implementation	158	1.41 (0.59–3.35)	1.17 (0.49–2.80)	1.48 (0.75–2.92)	1.46 (0.83–2.56)	1.94 (0.92–4.12)	2.21 (0.93–5.27)
Medium implementation	113	1.58 (0.66–3.83)	2.74 (1.09–6.92)	1.57 (0.78–3.16)	1.93 (1.01–3.67)	1.95 (0.90–4.22)	2.75 (1.10–6.86)
High implementation	66	0.53 (0.18–1.58)	0.65 (0.16–2.62)	0.50 (0.20–1.25)	0.52 (0.18–1.51)	0.65 (0.25–1.70)	0.95 (0.28–3.20)
* p-value*		0.077	0.066	0.026	0.061	0.016	0.068

^a^*n* = 1111

^b^Adjusted for sex, age, family occupational social class, school type (except stratified models), and baseline smoking status (models including smoking during school hours adjusted for baseline regular smoking)

^c^The categories were merged due to few cases in the low implementation group

### Differential Effects of Implementation Fidelity Across School Types

The associations between implementation fidelity and smoking outcomes differed when stratified by school types (Table 2). For students in social and healthcare schools, high implementation was associated with lower odds of smoking during school hours (OR 0.20, 95% CI 0.07–0.59). For students in technical, commercial, or preparatory schools, high implementation was not associated with smoking outcomes, but students in the medium implementation group had significantly higher odds of smoking daily (OR 2.74, 95% CI 1.09–6.92), regularly (OR 1.93, 95% CI 1.01–3.67), and during school hours (OR 2.75, 95% CI 1.10–6.86).

### Effects of The Separate Intervention Components on Smoking Outcomes

The impact of implementation of the tobacco policy, curriculum, and class competition components on the smoking outcomes was analyzed separately. High implementation of the tobacco policy was not significantly associated with the smoking outcomes though the estimates were in the intended direction, i.e., lower odds of smoking in the high implementation group (Table 3). Students in the low and medium implementation groups had higher odds of smoking outcomes though not significantly and most pronounced in the low implementation group. We detected no associations between implementation of the curriculum and competition components and smoking outcomes (Table 3).

**Table 3:** Odds ratios of smoking daily, regularly, and during school hours by low, medium, and high implementation of the intervention components (tobacco policy, curriculum, and competition) compared to students in the control group (n = 1,112)

		Daily smoking (*n* = 293)		Regular smoking (*n* = 348)		Smoking during school hours (*n* = 308)	
	*n*	OR (95% CI) Unadjusted	OR (95% CI) Adjusted^a^	OR (95% CI) Unadjusted	OR (95% CI) Adjusted^a^	OR (95% CI) Unadjusted	OR (95% CI) Adjusted^a^
**Control (ref)**	567	1	1	1	1	1	1
**School tobacco policy**^**b**^							
Low implementation	230	2.63 (1.19–5.82)	1.58 (0.73–3.39)	2.33 (1.19–4.56)	1.37 (0.70–2.68)	2.40 (1.28–4.50)	1.43 (0.67–3.13)
Medium implementation	97	0.98 (0.40–2.38)	1.06 (0.42–2.68)	1.00 (0.47–2.15)	1.13 (0.50–2.55)	1.16 (0.56–2.39)	1.30 (0.52–3.24)
High implementation	218	0.44 (0.19–1.01)	0.45 (0.19–1.08)	0.48 (0.24–0.98)	0.69 (0.33–1.42)	0.49 (0.25–0.96)	0.68 (0.30–1.58)
* p* Value		< 0.0001	0.024	< 0.0001	0.299	< 0.0001	0.168
**Educational curriculum**^**c**^							
Low implementation	195	1.00 (0.47–2.13)	0.68 (0.26–1.79)	1.18 (0.62–2.25)	1.12 (0.54–2.30)	1.31 (0.70–2.43)	1.05 (0.42–2.63)
Medium implementation	151	1.78 (0.86–3.70)	1.48 (0.57–3.88)	1.65 (0.88–3.11)	1.65 (0.80–3.40)	1.59 (0.86–2.92)	1.13 (0.45–2.85)
High implementation	199	0.99 (0.49–1.98)	0.92 (0.37–2.29)	0.88 (0.49–1.60)	0.72 (0.37–1.42)	0.99 (0.56–1.74)	0.93 (0.39–2.25)
* p* Value		0.112	0.350	0.111	0.217	0.284	0.965
**Class competition**^**d**^							
Low implementation	182	1.46 (0.73–2.91)	0.87 (0.35–2.14)	1.42 (0.79–2.55)	1.11 (0.54–2.27)	1.49 (0.85–2.61)	1.13 (0.50–2.58)
Medium implementation	182	1.39 (0.70–2.76)	1.07 (0.43–2.67)	1.22 (0.68–2.19)	1.01 (0.48–2.11)	1.26 (0.72–2.22)	1.03 (0.45–2.37)
High implementation	181	0.77 (0.38–1.57)	1.02 (0.38–2.72)	0.81 (0.45–1.49)	0.96 (0.43–2.14)	0.90 (0.51–1.62)	1.19 (0.49–2.88)
* p* Value		0.055	0.953	0.125	0.984	0.187	0.969

^a^Adjusted for sex, age, family occupational social class, school type, and baseline smoking status (models including smoking during school hours adjusted for baseline regular smoking because this outcome was not measured at baseline)

^b^Low 0–0%, medium 25.0–47.5%, and high 50.0–100% (tertiles of continuous variable)

^c^Low 0–12.5%, medium 25.0–25.0%, and high 50.0–100% (tertiles of continuous variable)

^d^Low 0–15.0%, medium 20.0–45.0%, and high 50.0–100% (tertiles of continuous variable)

### Additional Analyses

When decomposing implementation into school-level fidelity and student responsiveness, our additional analyses showed that both fidelity and responsiveness were associated with the smoking outcomes (Supplementary Table S2). Fidelity was associated with lower odds of regular smoking among all students and lower odds of smoking during school hours among social and healthcare vocational students similar to the observed associations of the main analyses. Among all students, the associations between positive attitudes towards smoke-free school hours and lower odds of smoking, and between negative attitudes and higher odds of smoking, did not remain significant after adjustment. Among social and healthcare vocational students, positive attitudes were associated with lower odds of smoking during school hours, and among students at the other school types, negative attitudes were associated with higher odds of all smoking outcomes.

We compared background characteristics of the included study population (*n* = 1112) with those of students excluded from analysis (Supplementary Table S3). A slightly higher proportion of the included students were from high socioeconomic position compared to students lost to follow-up (2 percentage points), and slightly more excluded students had low, unclassifiable, or missing data on socioeconomic status.

## Discussion

### Main Findings

This study investigated the association between implementation of the “Focus” smoking prevention intervention, i.e., the extent to which the intervention was delivered and received as intended, and smoking status and smoking during school hours after 4–5 months of intervention among Danish vocational and preparatory basic education students. We found that high levels of implementation were associated with lower odds of regular smoking and smoking during school hours, but not consistently with daily smoking. Stratified by school type, we found that high implementation was associated with lower odds of smoking during school hours in social and healthcare vocational schools, and no associations with decreased smoking in technical, commercial, and preparatory schools. On the contrary, a medium level of implementation was associated with higher odds of all smoking outcomes in these school settings. Lastly, we found that implementation of the intervention components was not associated with the smoking outcomes when analyzed separately.

### Comparison to Other Studies

In accordance with the present results, previous studies have demonstrated that the level of implementation matters for interventions’ effectiveness in general (Durlak & DuPre, 2008) and in smoking prevention interventions in elementary schools (Bast et al., 2016, 2019). To our knowledge, our study was the first to investigate the association between implementation of a smoking prevention intervention and smoking outcomes among vocational school students. Interestingly, the present study indicated a complex, non-linear relationship between level of implementation and smoking outcomes that differentiated between the school settings. A realist review by Schreuders et al. (2017) has similarly indicated that good quality implementation of STPs may lead to the intended effects of decreased smoking and that poor implementation could lead to reverse effects. Reverse effects have also been found in other studies (Poulin, 2007; Rozema et al., 2018). A Dutch study found that more adolescents started smoking at schools that implemented an outdoor school ground smoking ban compared to the control condition (Rozema et al., 2018). Schreuders et al. suggest mechanisms that either facilitate or counteract the impact of STPs on students’ smoking behavior, depending on the quality of the implementation process, in terms of enforcement practices, sanctioning, preventive efforts, etc. Examples of countervailing mechanisms include that students look for alternative places to smoke, develop new social meanings of smoking, undermine the harms of smoking, and alienate from school and the messages on smoking (Schreuders et al., 2017). Examples of facilitating mechanisms include that students feel they will get sanctioned for smoking, that students see less smoking during school hours and thereby feel less social pressure to smoke, and that students internalize anti-smoking beliefs (Schreuders et al., 2017). Similar countervailing and facilitating mechanisms activated in the implementation process might explain the complex relationship observed in the present study. Furthermore, such mechanisms might explain the variation across school types; the contextual conditions of the school types may have brought about different implementation processes that activated different mechanisms resulting in different outcomes. For example, low support of the tobacco policy and its enforcement from staff at technical vocational school could make students feel it was easy to avoid getting caught smoking and sensed a contradiction between the school’s anti-smoking message and actual enforcement practice. This could lead to students smoking at hidden locations instead of abstaining from smoking during the school day (Schreuders et al., 2017). Similarly, high support of the tobacco policy and its enforcement in line with an educational focus on health at social and healthcare vocational schools may have led to students internalizing the anti-smoking beliefs or believing they would get sanctioned for smoking (Schreuders et al., 2017). Students in social and healthcare schools may also be more motivated to quit or avoid smoking given their orientation towards health-related vocations. The Medical Research Council guidelines highlight that different overlapping research perspectives can be used in combination to explore the complexity of interventions, such as a theory-based perspective to inform an effectiveness evaluation (Skivington et al., 2021). Therefore, theory-based perspectives such as realist evaluation concerned with the interplay between context, mechanisms, and outcomes might be useful to further explore and unfold the complexities identified in the present study.

To our knowledge, few other studies have included component-specific implementation measurements in effectiveness evaluations of smoking prevention interventions (Bast et al., 2016, 2019). In the evaluation of a school-based smoking prevention intervention including, e.g., STP and curricular components, Bast et al. (2019) included number and combinations of components implemented and concluded that implementing all three intervention components mattered more for smoking prevention than implementing one or two components. Relatedly, our results indicate that high implementation of the overall intervention rather than the single intervention components affected students’ smoking behavior.

We found that overall implementation of the tobacco policy was not associated with our smoking outcomes. A longitudinal study among upper secondary school students across six European cities similarly found that a high total STP score considering enforcement, comprehensiveness, and communication, as reported from staff and students’ perspectives, was not associated with weekly smoking or smoking outside school premises but associated with less smoking on school premises (Mélard et al., 2020). However, the study also found that better enforcement of STPs was associated with less weekly smoking and less smoking on school premises (Mélard et al., 2020) indicating that enforcement is a significant aspect of implementing STPs in terms of changing students’ smoking behavior. We also found that implementation of the educational curriculum and class competition components did not affect the outcomes when analyzed separately, which is in line with the findings from Bast et al. (2019) concluding that exposure to curricular activities only, or any other single component, was not associated with less smoking (Bast et al., 2019). Using curricular materials and activities in school-based smoking prevention can be effective in preventing the onset of smoking and promoting attitude change (Mpousiou et al., 2021; Thomas et al., 2013). However, these interventions can be challenging to implement. As we observed in the “Focus” intervention, schools reported low levels of fidelity to the educational curriculum though students who received the sessions reported high acceptability (Jensen et al., 2023). Thus, we cannot conclude from this study that the educational curriculum or the class competition are redundant to the overall impact of the implementation on the outcomes. The intervention was designed to address multiple individual, interpersonal, and environmental determinants of students’ smoking behavior (Andersen et al., 2023) using a multi-component strategy (Coppo et al., 2014). In fact, it is recommended that STPs are accompanied by educational and preventive efforts to achieve the intended effects (Galanti et al., 2014; Schreuders et al., 2017). The unique contributions of the components on the smoking outcomes are difficult to detangle, and this task would require another study design such as a multi-arm trial allocating schools to implement single or different combinations of components. Instead, a possible interpretation of the findings is that the educational curriculum and class competition components are not essential to the effectiveness of the intervention, implicating that these components could be modified to fit local contextual conditions for scale-up or adaptation purposes without jeopardizing the outcomes (Blase & Fixsen, 2013).

### Methodological Considerations

A major strength of the present study was the measurement of implementation fidelity that was (1) underpinned by a comprehensive conceptual framework accounting for the implementation concepts' interaction (2) tailored to the specific program theory and assumptions of the “Focus” intervention, (3) component-specific which enabled analysis of how the intervention’s components influenced the outcomes, and (4) calculated as an individual-level construct that integrates school-level and student-level implementation. Individuals’ exposure and responsiveness are not always included as aspects of implementation fidelity (Moore et al., 2015). While some process evaluation or implementation frameworks incorporate whether and how participants come into contact with the intervention (i.e., “reach” or “coverage”) (Carroll et al., 2007; Linnan & Steckler, 2002), others view these as separate dimensions (RE-AIM; Glasgow et al., 1999) or as intervention outputs or intermediate outcomes (Moore et al., 2015), but there is no consensus on which to include (Moore et al., 2015). Moore et al. (2015) argue that evaluators should consider the extent to which participants’ engagement with the intervention is necessary to achieve effectiveness to determine whether reach should be included as an aspect of full implementation (Moore et al., 2015). More structural tobacco control interventions such as price policies could implicitly impact young people’s smoking behavior, but school tobacco policies, educational curricular activities, etc., require more conscious processes. Thus, in the case of “Focus”, we assume that the students would have to actively engage with the intervention components to achieve effectiveness, e.g., if students accept the policy to some extent, they will be more likely to comply with it. Furthermore, a key benefit of an individual-level measure is to avoid assuming that all students within the same school or unit are exposed equally to an intervention as it was delivered, thus considering each student’s uptake of the intervention (Ferm et al., 2018). Other strengths of the present study included the randomized study design enabling comparison of students’ smoking outcomes to a non-exposed control group, which has been requested in the literature on the effectiveness of STPs on students’ smoking to strengthen the evidence base (Galanti et al., 2014). The data collection took place during school hours to minimize non-response. The present study also has limitations. A limitation of the implementation construct is that we did not have appropriate data for all dimensions prescribed in the quantification model for all intervention components (Jensen et al., 2023). This implies that the intervention components’ constructs are not equally detailed and thus may vary in how well they capture the implementation process. Further, validated questionnaire items were not available from the literature. We used items from similar studies, where available, or developed new items tailored to the intervention components. We performed the analyses on a relatively small sample due to missing data which may have introduced type II error. Thus, the actual relationship between implementation of the intervention and students’ smoking might be stronger than estimated in this study. To assess potential selection bias, we compared background characteristics of the study population and students excluded from analysis. As differences were small, we concluded that selection bias was minimal, and we did not take further measures to handle missing data such as multiple data imputation. Another limitation is that the analyses that included smoking during school hours at follow-up were not adjusted for this variable at baseline. We did not include this item at baseline because the questionnaires were answered during the first weeks of the school semester, and many students would not have established a normal everyday school life at this point. Instead, we adjusted for baseline regular smoking because we assumed that students who smoked regularly at baseline would be likely to smoke during school hours at baseline.

### Implications

The findings of this study carry important implications for research and practice. Our study indicated enhanced effectiveness of the smoking prevention initiatives at high implementation levels and potential reverse effects at lower levels. A policy priority should therefore be to support the implementation process of school tobacco policies actively, e.g., through the employment of implementation strategies tailored to the school settings (Powell et al., 2015, 2017) as our findings also suggest that the context of implementation plays a role for the effectiveness. The additional analyses decomposing the impact of school-level fidelity and student responsiveness on students’ smoking outcomes indicate that strengthening schools’ implementation is important across all school types to reduce smoking. The analyses also strongly indicate that it is important to address students’ attitudes towards smoke-free policies in technical, commercial, and preparatory schools to avoid reverse effects. Among social and healthcare students, negative attitudes did not seem to play an important role for smoking behavior, but positive attitudes were associated with less smoking. Thus, improving school-level fidelity seems to be more important in this school type. Further research is needed to understand the mechanisms behind the differential effects observed in this study. We found that high implementation of the intervention decreased smoking during school hours. While a key task of smoking prevention is to keep young people from smoking at all, changing the visibility of smoking in the school environment is an important step towards sustainable change because it decreases the social acceptability of smoking (Coppo et al., 2014; Galanti et al., 2014), especially in school environments such as vocational schools with a persistent history of high smoking prevalence (Ringgaard et al., 2020).

This study was the first to apply the model for quantification of implementation developed by Ferm et al. (2018) in an effectiveness analysis. We found the model to be a feasible tool to assess implementation and its relation to individual outcomes. However, operationalizing the constructs required a wide range of questionnaire items which is often not feasible in large intervention studies. To lessen participant burden, it would be interesting to know whether more limited indicators could capture the relationship between implementation and outcomes. A simple school-level construct was used in the primary effectiveness study (Kjeld et al., 2023) categorizing students according to their schools’ delivery of either school and class level components (full intervention) or parts of the intervention only (partial intervention). The results showed that exposure to full intervention delivery, but not partial intervention delivery, affected daily smoking (OR: 0.44, 95% CI: 0.19–1.02) and regular smoking (OR: 0.57, 95% CI: 0.32–1.01) (Kjeld et al., 2023). Thus, using a simple construct of delivery can provide an indication of the relationship. This might be sufficient in some studies to complement an effectiveness study. However, to analyze the relationship between the variables more closely, more dimensions of implementation must be included and should be tailored to the assumptions underpinning the intervention (Ferm et al., 2018).

### Conclusions

From this study, we can conclude that high implementation fidelity of the “Focus” intervention was effective in reducing students’ regular smoking and smoking during school hours after 4–5 months. The relationship between implementation fidelity and students’ smoking was complex; high implementation raised the chances of decreased smoking while lower implementation could lead to reverse effects. These associations varied between the school types involved in the intervention. Thus, this study shows potential for enhancing and improving the quality of school-based smoking prevention among older adolescents and young adults. Our study adds to the knowledge base of the effectiveness of STPs on students’ smoking. It confirms that the existing evidence among younger age groups applies to school settings of older adolescents and young adults, i.e., that the implementation process is of key importance for interventions that include STPs to achieve their intended effects.

## Supplementary Information

Below is the link to the electronic supplementary material.

Supplementary file1 (PDF 168 KB)

Supplementary file2 (PDF 166 KB)

Supplementary file3 (PDF 138 KB)

Supplementary file4 (JPG 158 KB)

## Data Availability

The data used in this study are available from University of Southern Denmark (SDU). Restrictions apply to the availability of data under license for the current study and are therefore not publicly available. Data are available from the corresponding author upon reasonable request and with permission from SDU.
