# Supplementary material for: The Impact of Implementation Fidelity of a School-Based Multi-Component Smoking Prevention Intervention on Vocational Students’ Smoking Behavior: A Cluster-Randomized Controlled Trial
Source: Prev Sci. 2024 Aug 2;25(6):934–47. doi: 10.1007/s11121-024-01712-8 (PMC11390863; doi:10.1007/s11121-024-01712-8)
Supplement: Supplementary file 1 — Supplementary file1 (PDF 168 KB) [file 11121_2024_1712_MOESM1_ESM.pdf]

**Supplementary table 1**. Concepts and questionnaire items of implementation fidelity and success criteria (100 = optimally implemented, 50 = acceptably implemented, 0 = not implemented)			
**Implementation concept**	**Items**	**Success criteria**	**Data source**
**Smoke free school hours policy**			
Content	”Did the school introduce a smoke free school hours policy by the beginning of the school year in August?”	• All students and staff (100)<br>• All students but not staff, only a group of students, or other changes to the school tobacco policy the given school year, if relevant (50)<br>• No (0)	Principals
Quality (Information)	”How did you make new and current students, staff, and guests aware that the school had introduced a smoke free school hours policy?” Options: 1) held meeting between management and staff, 2) informed about the policy on school’s social media, information boards, webpage or likewise, 3) informed about the policy in classes or at a school gathering, 4) informed about the policy in a welcome letter or at information meeting before school start, 5) hung up posters from the “Focus” project, 6) put up signs that inform about the policy, 7) other (if relevant)	• At least two options chosen (100)<br>• One option chosen (50)<br>• No options chosen (0)	Principals
Quality (Dialogue)	”To which extent did you have an ongoing dialogue about the smoke free school hours policy since it was introduced? (e.g., talked about it in classes, mentioned it at meetings, written about on social media or school website) – Between students and employees”	• To a very high or high degree (100)<br>• To some degree (50)<br>• To a low degree or not at all (0)	Principals
Quality (Written policy)	”Did the school formulate a written policy for smoke free school hours?”	• Yes (100)<br>• No (0)	Principals
Quality (Enforcement by support and communication)	”This school year: What will typically happen if a student smokes during school hours?” Options: 1) A staff member has a talk with the student, 2) Principal/manager has a talk with the student, 3) The student is referred to the National Quit Line, 4) The student is referred to the staff member who attended the course in short conversations about smoking, 5) The student is referred to a mentor/student counsellor/coach, 6) The student is referred to other, e.g., municipal smoking cessation service	• At least one option chosen (100)<br>• No options chosen (0)	Principals
Quality (Frequent enforcement)	”In your impression: How often does the staff articulate the tobacco policy towards the students?”	• Daily or weekly (100)<br>• Monthly (50)<br>• Rarer or never (0)	Principals
Responsiveness	”It is fair that the school makes rules about whether we are allowed to smoke during school hours”	• Strongly agree or agree (100)<br>• Neither agree nor disagree (50)<br>• Disagree or strongly disagree (0)	Students
**Staff course**			
Content		• Two staff members participated (100)<br>• One staff member participated (50)<br>• No staff members participated (0)	Logbooks

Quality of delivery	”After the course: Did the management do any of the following to support that the participating staff could use their new knowledge and skills in practice?” Options: 1) Given one or more participating staff members disposable hours, e.g., to talk to students about smoking, 2) Allocated resources (e.g., time, finances) for participating staff to start new initiatives, 3) Held meeting with the participating staff members to talk about how they can put their new knowledge and skills into practice at school, 4) Encouraged other staff members to refer students in need of support or help in relation to smoking to the participating staff members, 5) Other, please state (if relevant).	• At least 1 option chosen (100)<br>• No options chosen (0)	Principals
	”The course has given me specific tools to talk to students about smoking in an open and motivating manner”	• Strongly agree or agree (100)<br>• Neither agree nor disagree (50)<br>• Disagree or strongly disagree (0)	Teachers
	”The course has given me more knowledge about young people, smoking, and addiction”	• Strongly agree or agree (100)<br>• Neither agree nor disagree (50)<br>• Disagree or strongly disagree (0)	Teachers
	”The course has given me a better understanding of the challenges and rewards of smoke free school hours”	• Strongly agree or agree (100)<br>• Neither agree nor disagree (50)<br>• Disagree or strongly disagree (0)	Teachers
	”After the course: Did you use your new knowledge and skills in practice at school?” Options: 1) Yes, I have had talks with students in need of help or support, 2) Yes, other staff members have referred students in need of help or support to me, 3) Yes, I have started new initiatives	• At least 1 option chosen (100)<br>• No options chosen (0)	Teachers
	”After the course: To which extent were you motivated to talk to students about smoking?”	• To a very high or high degree (100)<br>• To some degree (50)<br>• To a low degree or not at all (0)	Teachers
**Edutainment session**			
Content		• Edutainment session was held (100)<br>• Edutainment session was not held (0)	Logbooks
Quality of delivery	”In your impression: To which extent has the edutainment session promoted a dialogue, discussion, or reflection about own smoking and smoking at school in general?”<br>- Among school staff	• To a very high or high degree (100)<br>• To some degree (50)<br>• To a low degree or not at all (0)	Principals
	- Among the students	• To a very high or high degree (100)<br>• To some degree (50)<br>• To a low degree or not at all (0)	
Participation	”Did you participate in the edutainment session?”	• Yes (100)<br>• No (0)	Students
Responsiveness	”All in all, what did you think about the edutainment session?”	• Very good or good (100)<br>• Neither good nor bad (50)<br>• Bad or very bad (0)	Students
	”To which extent did the edutainment session make you think more about how much you smoke?”	• To a very high or high degree (100)<br>• To some degree (50)<br>• To a low degree or not at all (0)	

	”To which extent did the edutainment session make you think more about how smoking affects the body?”	• To a very high or high degree (100)<br>• To some degree (50)<br>• To a low degree or not at all (0)	
	”To which extent did the edutainment session make you talk more about smoking with other people?”	• To a very high or high degree (100)<br>• To some degree (50)<br>• To a low degree or not at all (0)	
	”To which extent did the edutainment session teach you something new about smoking that you did not already know?”	• To a very high or high degree (100)<br>• To some degree (50)<br>• To a low degree or not at all (0)	
**Educational curriculum**			
Content	“To your knowledge: To which extent has the material been used by the teachers at school?”	• To a very high or high degree (100)<br>• To some degree (50)<br>• To a low degree or not at all (0)	Principals
	Supplementarily (if missing or “I don’t know” on the above question): “Did you use the material?”	• At least one teacher answered yes (used all or parts of the material) (50)<br>• No teachers answered yes (0)	Teachers
Participation	”Did you do any of the following in class?” Options: 1) Made a policy for positive class culture, 2) Planned an activity for the class, 3) Talked about prejudices and common misperceptions of smoking, 4) Talked about activities to do during breaks, 5) Talked about health in internships and work life, 6) Worked with “attitude-cards” (e.g., to talk about attitudes towards smoking, health dilemmas, or well-being, loneliness, and school absence)	• Yes to at least three of the six activities (100)<br>• Yes to one or two of the six activities (50)<br>• Yes to none of the six activities (0)	Students
Responsiveness	”What did you think about using attitude cards in class?”	• Very good or good (100)<br>• Neither good nor bad (50)<br>• Bad or very bad (0)	Students
**Quit-and-win competition**			
Content		• Students’ carbon monoxide levels were measured in the classes (100)<br>• Students’ carbon monoxide levels were not measured in the classes (0)	Logbooks
Participation	”Were carbon monoxide measurements conducted in your class?”	• Yes (100)<br>• No (0)	Students
	”Did you get your carbon monoxide level measured?”	• Yes, both times (100)<br>• Yes, one time (50)<br>• No, not present (0)	
Responsiveness	“All in all, what did you think about the class competition with carbon monoxide measurements?”	• Very good or good (100)<br>• Neither good nor bad (50)<br>• Bad or very bad (0)	Students
	“The competition made me think about how smoking affects the body”	• Strongly agree or agree (100)<br>• Neither agree nor disagree (50)	

		• Disagree or strongly disagree (0)	
	"The competition made me want to smoke less or avoid to start smoking"	• Strongly agree or agree (100)<br>• Neither agree nor disagree (50)<br>• Disagree or strongly disagree (0)	
	"The competition gave me a sense of cohesion in class"	• Strongly agree or agree (100)<br>• Neither agree nor disagree (50)<br>• Disagree or strongly disagree (0)	
	"We helped and supported each other to smoke less to win the competition."	• Strongly agree or agree (100)<br>• Neither agree nor disagree (50)<br>• Disagree or strongly disagree (0)	
**Access to smoking cessation support**			
Quality of delivery	"Did the school do any of the following to inform students and staff about the smoking cessation offer from the National Quit Line?" Options: 1) Hung up the mailed posters from the National Quit Line at school, 2) Mentioned the offer for students at gatherings or in class, 3) Mentioned the offer at meetings with management and staff, 4) Mentioned the offer on school's social media or homepage, 5) Mentioned the offer in material for new student, e.g., welcome letter, 6) Encouraged staff to inform students about the offer	• At least two options chosen (100)<br>• One option chosen (50)<br>• No options chosen (0)	Principals
	Supplementarily (if missing in the question above): "Did you do any of the following to raise awareness of the smoking cessation offer from the National Quit Line?" Options: 1) Mentioned it for students in my class, 2) Mentioned it for students outside class, 3) Mentioned it for colleague(s)	• At least one teacher chooses at least two options (100)<br>• At least one teacher chooses one option (50)<br>• No teachers choose an option (0)	Teachers
Participation	"Do you know about this offer?" (Item accompanied by a picture of the National Quit Line poster)	• Yes (100)<br>• No (0)	Students
Note: When there is more than one measure of the same implementation concept, we calculated a mean as the overall measure			
