# Supplementary material for: The Impact of Implementation Fidelity of a School-Based Multi-Component Smoking Prevention Intervention on Vocational Students’ Smoking Behavior: A Cluster-Randomized Controlled Trial
Source: Prev Sci. 2024 Aug 2;25(6):934–47. doi: 10.1007/s11121-024-01712-8 (PMC11390863; doi:10.1007/s11121-024-01712-8)
Supplement: Supplementary file 2 — Supplementary file2 (PDF 166 KB) [file 11121_2024_1712_MOESM2_ESM.pdf]

Supplementary table 2. Additional Analyses. Odds Ratios of Smoking Daily, Regularly, and During School Hours by School's Fidelity Level (low, medium, high[a]) of the Total Intervention Compared to the Control Group

		Daily smoking		Regular smoking		Smoking during school hours	
	n	OR (95% CI)	OR (95% CI)*	OR (95% CI)	OR (95% CI)*	OR (95% CI)	OR (95% CI)*
**SCHOOL-LEVEL FIDELITY**							
All students	1112						
Control (ref)	567	1	1	1	1	1	1
Low fidelity	155	1.27 (0.46-3.49)	1.84 (0.55-6.14)	1.34 (0.58-3.09)	1.87 (1.06-3.30)	1.58 (0.80-3.10)	2.32 (1.00-5.36)
Medium fidelity	237	1.14 (0.47-2.74)	0.80 (0.27-2.43)	1.14 (0.55-2.36)	1.16 (0.65-2.06)	1.30 (0.72-2.34)	1.07 (0.50-2.28)
High fidelity	153	1.16 (0.42-3.21)	0.53 (0.13-2.11)	0.94 (0.40-2.20)	0.37 (0.18-0.77)	0.85 (0.42-1.72)	0.40 (0.15-1.09)
p-value		0.9683	0.5603	0.8866	0.0070	0.4070	0.0602
Social and health care students	415						
Control (ref)	207	1	1	1	1	1	1
Low/medium[c] fidelity	55	1.79 (0.98-3.28)	0.49 (0.04-6.52)	1.80 (0.86-3.77)	1.52 (0.27-8.46)	1.48 (0.81-2.71)	0.55 (0.12-2.54)
High fidelity	153	0.76 (0.48-1.20)	0.45 (0.06-3.42)	0.66 (0.37-1.19)	0.34 (0.10-1.23)	0.61 (0.38-0.96)	0.31 (0.10-0.96)
p-value		0.0305	0.7163	0.0370	0.1428	0.0137	0.1277
Technical, commercial, and preparatory students	697						
Control (ref)	360	1	1	1	1	1	1
Low fidelity	155	1.64 (0.58-4.61)	1.94 (0.63-5.98)	1.66 (0.76-3.59)	1.69 (0.97-2.94)	1.96 (0.97-3.96)	2.40 (1.00-5.75)
Medium/high fidelity	182	0.97 (0.34-2.75)	1.15 (0.33-4.08)	0.98 (0.45-2.13)	1.09 (0.57-2.08)	1.33 (0.66-2.67)	1.39 (0.55-3.55)
p-value		0.5913	0.5150	0.3824	0.1754	0.1726	0.1433
**STUDENT RESPONSIVENESS[b]**							
All students	1112						
Control (ref)	567	1	1	1	1	1	1
Negative attitudes	163	2.99 (1.61-5.54)	1.69 (0.71-4.03)	2.73 (1.60-4.64)	1.65 (0.80-3.42)	2.76 (1.72-4.44)	1.63 (0.74-3.59)
Neutral	177	1.11 (0.59-2.09)	0.86 (0.36-2.06)	1.09 (0.64-1.87)	0.92 (0.45-1.88)	1.37 (0.85-2.19)	1.27 (0.59-2.74)
Positive attitudes	205	0.38 (0.19-0.77)	0.57 (0.22-1.45)	0.44 (0.25-0.79)	0.73 (0.35-1.54)	0.44 (0.26-0.74)	0.61 (0.27-1.37)
p-value		<0.0001	0.0571	<0.0001	0.1530	<0.0001	0.0207
Social and health care students	415						
Control (ref)	207	1	1	1	1	1	1
Negative attitudes	65	2.53 (1.22-5.29)	0.75 (0.15-3.87)	2.18 (0.88-5.44)	0.54 (0.10-3.12)	1.95 (0.92-4.13)	0.55 (0.17-1.75)
Neutral	67	0.91 (0.43-1.93)	0.41 (0.08-2.19)	0.86 (0.34-2.17)	0.54 (0.09-3.17)	0.80 (0.37-1.72)	0.40 (0.12-1.31)
Positive attitudes	76	0.30 (0.13-0.72)	0.30 (0.05-1.71)	0.31 (0.12-0.84)	0.51 (0.08-3.21)	0.22 (0.09-0.55)	0.19 (0.05-0.71)
p-value		<0.0001	0.4184	<0.0001	0.8938	<0.0001	0.0925
Technical, commercial, and preparatory students	697						
Control (ref)	360	1	1	1	1	1	1
Negative attitudes	98	3.20 (1.45-7.06)	2.66 (1.06-6.68)	3.05 (1.63-5.71)	2.62 (1.34-5.11)	3.41 (2.00-5.81)	2.69 (1.28-5.65)
Neutral	110	1.21 (0.54-2.72)	1.31 (0.52-3.28)	1.24 (0.66-2.33)	1.19 (0.62-2.27)	1.88 (1.11-3.16)	2.20 (1.08-4.45)
Positive attitudes	129	0.42 (0.17-1.04)	0.87 (0.31-2.40)	0.54 (0.27-1.06)	0.92 (0.46-1.85)	0.63 (0.36-1.12)	1.05 (0.50-2.22)
p-value		<0.0001	0.0976	<0.0001	0.0332	<0.0001	0.0111

[a]Low: 0-72.3%, medium: 82.6-86.3%, and high: 88.8-100% (tertiles of continuous variable), *Adjusted for sex, age, SES, school type, and baseline smoking status, [b] Responsiveness of the smoke-free school hours component (Agreement to the statement: "It is fair that the school makes rules about smoking during school hours". Totally agree or agree (positive attitudes), neither agree nor disagree (neutral), disagree or totally disagree (negative attitudes).
