# Supplementary material for: The Impact of Implementation Fidelity of a School-Based Multi-Component Smoking Prevention Intervention on Vocational Students’ Smoking Behavior: A Cluster-Randomized Controlled Trial
Source: Prev Sci. 2024 Aug 2;25(6):934–47. doi: 10.1007/s11121-024-01712-8 (PMC11390863; doi:10.1007/s11121-024-01712-8)
Supplement: Supplementary file 3 — Supplementary file3 (PDF 138 KB) [file 11121_2024_1712_MOESM3_ESM.pdf]

Supplementary table 3.

Characteristics of the Study Population and Students Excluded From Analysis

	Students included in the present study (N=1112)	Students lost to follow-up, i.e., implementation % not calculated (N=806)	Students with limited implementation data (N=61)
School type			
Social and healthcare	415 (37.3)	262 (32.5)	-
Technical and commercial	534 (48.0)	309 (38.3)	-
Preparatory basic	163 (14.7)	235 (29.2)	61 (100)
Age, years (median)	17	18	19
Sex			
Female	558 (50,2)	400 (49.6)	26 (42.6)
Male	554 (49.8)	406 (50.4)	25 (41.0)
Missing	-	-	10 (16.4)
Family occupational social class			
High (I+II)	165 (14.8)	104 (12.9)	2 (3.3)
Middle (III+IV)	449 (40.4)	325 (40.3)	19 (31.2)
Low (V+VI)	265 (23.8)	210 (26.1)	19 (31.2)
Unclassifiable	211 (19.0)	157 (19.5)	10 (16.4)
Missing	22 (2.0)	10 (1.2)	11 (18.0)
