# Supplementary figures and images for: The Impact of Implementation Fidelity of a School-Based Multi-Component Smoking Prevention Intervention on Vocational Students’ Smoking Behavior: A Cluster-Randomized Controlled Trial
Source: Prev Sci. 2024 Aug 2;25(6):934–47. doi: 10.1007/s11121-024-01712-8 (PMC11390863; doi:10.1007/s11121-024-01712-8)

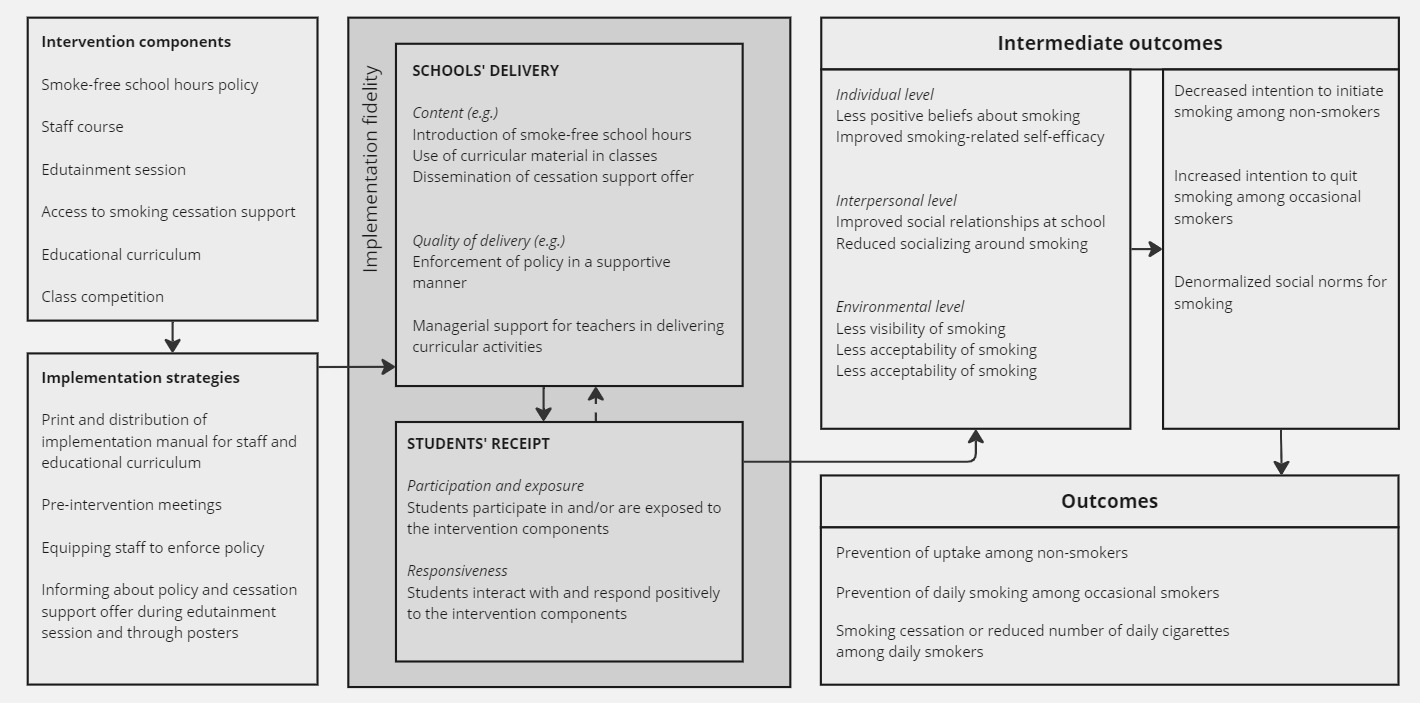

Supplement: Supplementary file 4 — Supplementary file4 (JPG 158 KB) [file 11121_2024_1712_MOESM4_ESM.jpg]
